# Chicken feet reimagined: A strategic platform for nutritional innovation, industrial valorization, and academic-industry synergies

**DOI:** 10.1016/j.psj.2026.106888

**Published:** 2026-04-01

**Authors:** Farid S. Nassar

**Affiliations:** Department of Animal and Fish Production, College of Agricultural and Food Sciences, King Faisal University, Al-Ahsa, Saudi Arabia

**Keywords:** Chicken feet, Collagen and gelatin, Functional food, Poultry byproducts, Sustainability

## Abstract

Chicken feet are an underutilized poultry by-product despite their richness in collagen, essential minerals, and bioactive peptides, which highlights a clear gap between their intrinsic value and current industrial utilization. This study contributes to developing an integrated, multi-dimensional strategic framework that reconceptualizes chicken feet as a high-value resource linking nutritional innovation, industrial valorization, and sustainability in food production systems. Methodologically, the study adopts a qualitative descriptive design supported by a systematic review conducted in accordance with Systematic Reviews and Meta-Analyses (PRISMA) guidelines, integrating evidence from peer-reviewed literature, international reports, and global case studies to ensure methodological rigor and analytical depth. The findings demonstrate that chicken feet represent a sustainable and competitive source of high-quality collagen and gelatin, characterized by valuable functional, nutritional, and bioactive properties, making them suitable for applications in food, health, and bio-based packaging industries. The primary novelty of this study lies in bridging fragmented research domains by integrating nutritional science, industrial processing, and sustainability into a unified, implementation-oriented framework. This framework is operationalized through interconnected stages encompassing knowledge generation, technological development, industry integration, capacity building, and the sustainable utilization of resources. Furthermore, the study highlights the strategic role of poultry science programs in higher education as innovation hubs capable of transforming scientific knowledge into marketable products through effective academic–industry collaboration. Practical implications include improving resource efficiency, reducing waste, supporting sustainable production systems, and creating new economic opportunities in global markets. The study concludes by positioning chicken feet as a key component of a sustainable bio-based economy and provides evidence-based recommendations for policymakers, researchers, and industry stakeholders to maximize their economic, nutritional, and functional value.

## Introduction

Poultry production sciences play a strategic role in enhancing food security and meeting the rising global demand for animal protein, while contributing to sustainability (Nassar, 2026). Based on current global trends, global poultry meat production is expected to continue increasing at a steady rate, rising by 2% in 2025 to reach approximately 105.8 million tons (USDA, 2025a). Estimates further indicate that global consumption will reach around 173 million tons of ready-to-cook poultry products by 2034, representing approximately 62% of the total growth in global meat consumption, with poultry projected to provide about 45% of total meat-derived proteins by the same year (OECD/FAO, 2025). This expansion is attributed to lower poultry production costs, favorable protein-to-fat ratios, high nutritional value, and a lower environmental footprint compared to red meats, making it a preferred option for sustainability- and cost-conscious consumers.

However, this rapid growth in production is accompanied by the generation of substantial quantities of by-products. Secondary products, both edible and non-edible, can account for up to 30% of total production, which may pose increasing environmental and economic challenges (Talha et al., 2024). The poultry production system encompasses all stages from chick rearing to slaughter, generating massive amounts of waste, including litter, feathers, bones, and unfit carcasses, estimated at approximately 68 billion tons annually (McGauran et al., 2021).

Amid global challenges related to food security, scarce land and water resources, climate change, and population growth, adopting sustainable food systems has become imperative. Consumers are increasingly aware of the importance of transparency and sustainability in food supply chains (Radhakrishnan et al., 2020). In this context, the concept of a circular economy emerges as a strategic framework for repurposing waste and transforming it into value-added products within a sustainable production and consumption system (Chiarelotto et al., 2021). Efficient management of poultry slaughter by-products is a pivotal step toward making the sector more productive and environmentally friendly (Talha et al., 2024).

Poultry industry by-products also represent promising sources for extracting bioactive molecules that can be utilized as high-value components in various industries, including food, pharmaceutical, and biomedical sectors (Ozturk-Kerimoglu et al., 2023). Compositional analyses have shown that slaughter by-products such as feathers, skin, feet, heads, and trachea contain approximately 34.2% dry matter, consisting of 50–63% protein and 9–15.5% ash (Kazemi-Bonchenari et al., 2017). It is estimated that 7–8% of broiler chicken weight comprises cut parts and bones, equivalent to about 9.5 million tons of secondary products annually that are suitable for protein extraction (Salminen and Rintala, 2002).

These by-products are rich in structural proteins; blood, viscera, feet, and bones contain high proportions of collagenous and gelatinous proteins (Bichukale et al., 2018). Historically, these proteins were extracted from Porcine (46%), cowhide (29.4%), and pork and cattle skeletons (23.1%) (Alipal et al., 2021). However, the emergence of zoonotic diseases such as Bovine Spongiform Encephalopathy (BSE) and Foot-and-Mouth Disease (FMD), along with their potential risks to human health, in addition to cultural and religious constraints, has driven research efforts toward the exploration and development of safe alternative sources for gelatin production (Abedinia et al., 2017).

In this regard, chicken feet emerge as one of the most promising by-products. Given their relatively high protein content, particularly collagen, they represent an effective raw material for producing high-value gelatin (Bravo et al., 2023). Chicken feet consist of skin, bones, muscles, and connective tissues, with collagen constituting approximately 30–35% of their composition (Kuan et al., 2017). Collagen from chicken feet is characterized by a rich amino acid profile, including glycine (35%), alanine (11%), and relatively high levels of proline (Almeida and Lannes, 2013). Extracted collagen serves as a key source for gelatin used in food industries as a gelling agent, moisture stabilizer, thickener, and for food packaging applications (Li et al., 2007; Moraes and Cunha, 2013; Araújo et al., 2019; Afidah, 2025).

Moreover, chicken feet have traditionally been used as a food ingredient in several Asian and African countries and have recently been developed into commercially available ready-to-eat products, especially in China, where the edible portion’s high connective tissue content provides unique textural properties in terms of chewiness and elasticity, contributing to consumer preference (Ratna et al., 2023; Tang et al., 2023; Jia et al., 2024). Additionally, chicken feet can be used to produce protein hydrolysates with angiotensin-converting enzyme inhibitory activity (ACEi hydrolysates), further enhancing their functional and health value (Bravo et al., 2023).

Therefore, repurposing chicken feet within a circular economy framework not only provides an environmental solution to reduce waste but also opens wide avenues for developing high-value functional and nutritional components, contributing to the economic and environmental sustainability of the poultry industry, while aligning with global trends toward more sustainable production and consumption systems. The objective of this study is to reconceptualize chicken feet, transforming them from a low-utilized by-product into a multi-dimensional strategic resource by developing an integrated framework that links scientific research with industrial applications and food innovation, thereby enhancing sustainability and maximizing their economic and functional value within sustainable food production systems. The study aims to address four key aspects: first, identifying the nutritional and health value of chicken feet and their significance for humans; second, exploring their importance as a sustainable source of high-value collagen and gelatin; third, establishing a strategic framework for universities to utilize chicken feet as a promising resource and analyzing the implications of its implementation; and fourth, identifying challenges related to the sustainability and utilization of chicken feet in local and global markets, thus enabling the exploration of innovation opportunities and achieving sustainable economic and industrial value from these vital secondary products.

Chicken feet (also known as paws, claws, or the distal lower limbs) represent a resource rich in collagen, gelatin, and bioactive compounds; however, their utilization remains limited and largely traditional, without fully harnessing their nutritional and industrial potential. Literature analysis reveals a scarcity of applied and integrative studies in this field, particularly regarding comparisons of the industrial performance of chicken-feet-derived gelatin with conventional sources, biosafety assessment, and industrial scalability across diverse contexts. This research gap highlights the emerging and promising nature of the field, offering substantial opportunities for universities and research and industrial institutions to develop sustainable functional products, enhance nutritional and industrial value, and explore new markets. Moreover, existing studies have predominantly focused on isolated aspects such as nutritional composition or extraction techniques, lacking an integrative framework that combines scientific research, industrial applications, food innovation, safety and quality assurance, and education. This fragmentation underscores a significant scientific gap that limits the transformation of chicken feet into a strategic resource for sustainable food and industrial innovation, emphasizing the need to develop a multidimensional framework to advance research, innovation, and future applications in the food and health sectors.

In this context, this study provides several key contributions to enhancing the added value of chicken feet as one of the poultry by-products and strengthening sustainable food systems through the development of an integrated, multi-dimensional strategic framework that reconceptualizes chicken feet as a high-value resource linking nutritional innovation, industrial valorization, and sustainability in food production systems; adopting a qualitative descriptive methodology supported by a systematic review conducted in accordance with Systematic Reviews and Meta-Analyses (PRISMA) guidelines, thereby ensuring methodological rigor and comprehensive synthesis of the available evidence; bridging fragmented research domains by integrating insights from nutritional science, industrial processing, and sustainability into a unified, implementation-oriented framework; demonstrating the functional, nutritional, and bioactive potential of chicken feet as a sustainable source of high-quality collagen and gelatin with applications in food, health, and bio-based packaging industries; proposing an operational framework encompassing knowledge generation and research, technology development, industry integration, capacity building, and sustainable resource utilization; highlighting the strategic role of poultry science programs in higher education as innovation hubs that facilitate university–industry collaboration and support the translation of research outputs into marketable products; and providing practical and policy-oriented recommendations to enhance resource efficiency, reduce waste, and support the sustainable development of the poultry industry.

## Material and methods

**Fig. 1:**
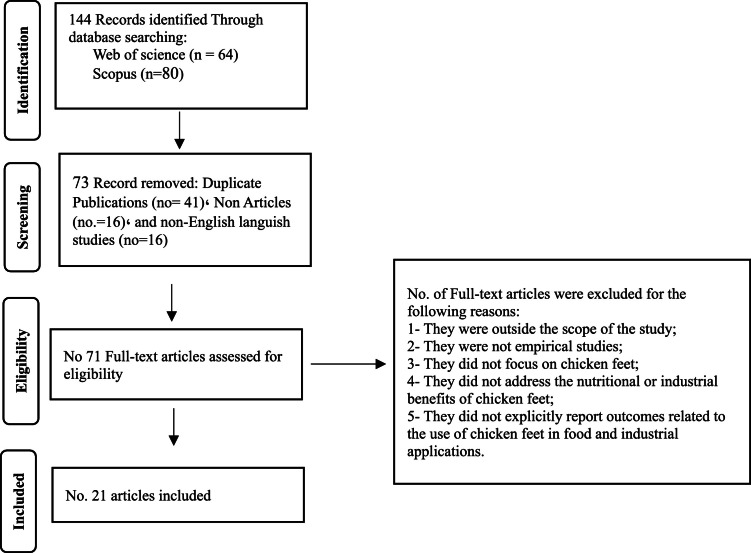
PRISMA flowchart of study selection and inclusion process.

This study employs a qualitative descriptive research design to reconceptualize chicken feet as a high-value strategic resource through an integrated framework that advances food innovation, industrial valorization, and sustainability in food production systems. The study followed the PRISMA guidelines (Liberati et al., 2009; Moher et al., 2015) due to their capacity to enhance transparency, minimize bias, and ensure a systematic and traceable approach to conducting systematic reviews (Afrifa et al., 2022)., with the methodological steps clearly illustrated in Figure 1. To enhance transparency and methodological rigor, the manuscript has been revised to provide a comprehensive PRISMA flow description. A total of 144 records were initially identified through database searching, including Web of Science (*n* = 64) and Scopus (*n* = 80). Following the initial screening, 73 records were excluded, comprising duplicate publications (*n* = 41), non-article documents (*n* = 16), and non-English language studies (*n* = 16). Subsequently, 71 full-text articles were assessed for eligibility, of which 21 studies met the predefined inclusion criteria and were ultimately included in the review, as clearly presented in Figure 1.

In the first phase, a systematic and comprehensive search was conducted across leading academic databases, including Scopus and Web of Science, to identify scientific sources related to the role of chicken feet as a high-value by-product. The study employed a targeted set of keywords such as “Chicken Feet”, “Poultry Production”, “Poultry Industry”, “Nutritional Value”, “Functional Food”, “Collagen and Gelatin”, and “Poultry Science Programs”. The search focused on peer-reviewed articles, institutional reports, and empirical studies published in English between the years 2021 and 2025, supplemented by foundational literature to establish a robust theoretical framework supporting the strategic utilization of chicken feet in food and industrial production.

In the second phase, strict inclusion and exclusion criteria were established to ensure the accuracy and relevance of sources. Studies were included if they directly addressed the nutritional and health significance of chicken feet for humans, as well as their contribution to the development of sustainable food and industrial products, with a focus on production and manufacturing sustainability. Studies that lacked a clear connection to the study topic, higher education, or poultry production sciences, or that did not meet rigorous methodological or empirical standards, were excluded.

In the third phase, data were extracted and analyzed qualitatively and thematically. The analysis focused on each study’s objectives and findings regarding the nutritional and health value of chicken feet and their potential for functional product development. It also examined factors affecting sustainability and production efficiency, aiming to determine how university poultry science programs can leverage these insights to develop innovative and sustainable production systems.

In the fourth phase, findings were synthesized within an integrated strategic framework that enables poultry science programs to strengthen their role as specialized educational programs in developing functional and food products, while accelerating the adoption of scientific and technological innovations. This phase emphasized academic initiatives, training programs, research and technical capacity development, and industry partnerships, empowering higher education institutions to transform chicken feet into high-value food, functional, and industrial products, while promoting sustainable innovation and fostering integration between academic research and industrial application.

Furthermore, throughout all phases of information handling, sources were rigorously screened to ensure scientific relevance and systematically organized using a structured information-extraction framework, including study title, abstract, results, and key conclusions. Qualitative thematic analysis was then applied to identify recurring patterns, main themes, and emerging trends, with findings synthesized and interpreted in alignment with the study’s conceptual framework and strategic objectives, ensuring methodological rigor and full academic reliability.

By integrating descriptive, interpretative, and strategic elements within this structured four-phase methodology, the study not only provides a rigorous and comprehensive assessment of the role of chicken feet as a high-value by-product but also ensures analytical synthesis, methodological depth, and originality in the findings. This framework addresses previous limitations, offering evidence-based strategies to maximize the nutritional and industrial benefits of chicken feet, enhance sustainability, and maintain the credibility and relevance of the research.

## Results and discussion

### Nutritional and health value of chicken feet and their significance for humans

Chicken feet represent one of the most prominent by-products in broiler chicken production, yet their consumption varies significantly across countries depending on cultural patterns and dietary traditions. In 2021, China ranked second globally in broiler chicken production; however, China and a limited number of other Asian countries are among the few nations that widely consume chicken feet, due to deeply rooted cultural factors (Yuan et al., 2023). In contrast, in many Western countries, such as European nations and the United States, chicken feet are classified as underutilized by-products, reflecting a clear gap between production and consumption. Trade data highlight the scale of this gap: China imported approximately 491,000 tons of frozen chicken feet during the first eleven months of 2023 to meet domestic demand, according to the General Administration of Customs of China (Tang et al., 2024). Market reports indicate that strong demand is expected to persist until 2026, despite trade restrictions related to highly pathogenic avian influenza (HPAI) and export challenges in some exporting countries (USDA, 2025b). Chicken feet account for approximately 60–75% of China’s annual imports of poultry products, underscoring their economic and nutritional significance in the Asian market (Tridge, 2020).

Chicken feet are composed of skin, bones, muscles, and connective tissues and are characterized by a high protein content, particularly collagen, ranging from 18 to 23 g per 100 g of wet weight (Bravo et al., 2023)**.** The detailed nutritional composition of various poultry by-products, including chicken feet, bones, feathers, blood, skin, and cartilages, illustrates protein, moisture, fat, and mineral content for each product, as presented in Table 1 (Bravo et al., 2023)**.** Collagen is a structural protein rich in distinctive amino acids, forming the basis for gelatin and bioactive peptides production. In addition to protein content, chicken feet are an important source of essential minerals (Bravo et al., 2023)**.**

**Table 1:** Composition of chicken byproducts (%west basis) (Bravo et al., 2023).

Chicken by-products	Protein %	Ach %	Moisture %	Fat %	Carbohydrate%	Mineral%	Fiber%
**Feet**	18.10-22.91	7.94-8.16	58.02-65.08	3.90-6.2	-	-	-
**Bones**	15.6-23.38	11.80-12.35	53.2-57.54	8.40-9.52	1	14.7-15.9	-
**Feathers**	80-85.31	0.69-0.83	2.03-10.06	2-3.92	-	1.2	˂1
**Blood**	14.46-20.55	0.81-3.74	75-82	0.033-1.69	0.363	-	-
**Skin**	8.5-15.21	0.64-0.9	43.70-54.22	31.44-41.4	-	-	-
**Cartilages**	11.78	1.21	82.85	0.29	3.87	-	-

Juliani et al., 2023a, stated that nutritional analyses of prepared chicken feet products, such as porridge produced through boiling and grinding, revealed that per 100 g, they contain approximately 10.4% protein, 611.2 mg calcium, 0.85 mg iron, 1.08 mg zinc, and 284.5 mg phosphorus, making them an effective contributor to meeting the calcium requirements of pregnant women and supporting bone health (Juliani et al., 2023a). An interventional dietary study demonstrated that consuming 100 g per day of processed chicken feet for one month significantly increased calcium levels in adolescent girls’ blood by approximately 1.1 mg/dL, an effect roughly equivalent to taking a 500 mg daily calcium supplement, indicating their potential as a dietary source supporting bone health (Mulyani et al., 2024).

Beyond their traditional nutritional value, chicken feet are a rich source of bioactive peptides with multiple functional activities. Studies have shown that collagen extracted from chicken feet can yield peptides with antioxidant, antidiabetic, lipid-lowering, immunomodulatory, and anti-aging properties (Fu et al., 2019; Cao et al., 2020, 2022). Other studies confirmed that they serve as an excellent source of antioxidant peptides, while further research is needed to understand their bioavailability in humans and potential industrial applications (Ozturk-Kerimoglu et al., 2023). In the context of joint health, a recent study demonstrated that combining chicken feet extract with traditional medicinal herbs helped alleviate osteoarthritis symptoms, reduce cartilage degradation, and lower inflammatory responses, highlighting their potential as a functional or supplemental dietary component for musculoskeletal health (Beak et al., 2025). Regarding cardiovascular health, animal studies indicated that proteins extracted from chicken feet can be converted into peptides capable of inhibiting angiotensin-converting enzyme, improving vascular function, and increasing antioxidant levels in the body, mechanisms that contribute to blood pressure reduction (Bravo et al., 2023).

The use of chicken feet extends beyond traditional consumption to the development of high-value food products. Hadi et al. (2022) demonstrated that the addition of chicken feet flour to the crispy corn product significantly enhanced its nutritional value, reflected by increases in energy, protein, fat, moisture, and ash content. Additionally, s study on a nutritional formulation composed of chicken feet and mung bean in a 2:1 ratio showed high sensory acceptance and compliance with complementary food standards for pregnant women at risk of chronic energy deficiency, highlighting its potential for developing high-protein, high-calcium foods (Juliani et al., 2023b). The proliferation of deboned chicken feet products reflects a shift in consumption patterns toward more convenient and ready-to-eat products, particularly in fast food and snack markets (Tang et al., 2024). Understanding consumer behavior thus becomes a strategic tool enabling producers and policymakers to translate these insights into innovative and sustainable production practices that enhance competitiveness and meet future demand while maintaining a balance between profitability, quality, and social and environmental responsibility (Nassar, 2026).

Chicken feet have also emerged as a functional ingredient in meat product formulation. Results from Miwada et al. (2025a) indicated that adding 5–15% chicken foot skin to sausages improved nutritional value and antioxidant capacity due to its high collagen content. Another study (Miwada et al., 2025b) confirmed that this addition enhanced protein content and sensory properties, including taste and aroma. In another application, collagen gel extracted from chicken feet was used as a fat substitute in chicken sausages, yielding products with higher water retention, better resistance to lipid oxidation, and improved cardiovascular health indices, while maintaining sensory attributes during 42 days of refrigerated storage (Araújo et al., 2021).

Processing methods directly influence the physicochemical properties and quality of chicken feet. A study by Yuan et al. (2023) highlighted that the thawing method plays a crucial role in maintaining the quality of frozen feet, with ultrasonic thawing considered the most suitable option for both domestic and industrial settings, providing a balance between product quality preservation and physicochemical safety. From a microbiological perspective, Jia et al. (2024) investigated spoilage causes in ready-to-eat chicken feet, opening avenues for more effective spoilage control strategies and ensuring consistent quality, while emphasizing the need for further research to understand spoilage mechanisms, particularly the relationship between microorganisms and volatile compounds associated with chicken feet products. In terms of packaging and storage, Chen et al. (2025) compared air packaging and vacuum packaging for chicken feet processed using the “tiger skin” method, showing that vacuum packaging provided better stability regarding total bacterial colony counts and sensory evaluation, indicating its efficiency in maintaining quality, along with a recommendation to develop intelligent packaging systems and modern preservation technologies to enhance safety and quality. In this context, Zhang et al. (2024) indicate that the development of advanced biosensors for rapid and noninvasive bacterial detection represents a promising approach to supporting intelligent packaging systems. Accordingly, these technologies can be employed to enhance the packaging and storage systems of chicken feet by integrating them into packaging materials or monitoring systems, enabling the early detection of microbial contamination during storage and transportation, as well as real-time monitoring of product quality. This contributes to ensuring product safety, preserving sensory and nutritional attributes, and reducing product losses. Regarding physical processing, Tang et al. (2024) demonstrated that optimizing expansion processing in deboned chicken feet enhances water-holding capacity, boosting market value and creating clear economic significance.

Ultimately, chicken feet are no longer merely a low-value by-product but represent a multi-dimensional nutritional and functional resource. Nutritionally, they provide a rich source of protein, calcium, and essential minerals, making them suitable for supporting bone health, particularly among sensitive populations such as adolescents and pregnant women. Functionally, bioactive peptides derived from chicken feet open new avenues for developing functional foods and dietary supplements with antioxidant, anti-inflammatory, and blood pressure-lowering effects. Economically, strong demand in Asian markets, especially China, reflects significant commercial value that remains underutilized in many poultry-producing countries. Continued reliance on imports highlights a gap in value chains that can be addressed by developing local processing technologies to convert chicken feet into high-value food or functional products. Therefore, reevaluating chicken feet from nutritional, health, and economic perspectives represents a strategic step toward maximizing poultry by-product utilization, transforming them into a sustainable food resource that supports food security, enhances public health, and generates added value for the industry simultaneously.

### Importance of chicken feet as a sustainable source of collagen and gelatin

Gelatin is one of the most widely used hydrocolloids across the food, pharmaceutical, cosmetic, and packaging industries due to its foaming, emulsifying, and gelling properties, as well as its ability to form films (Rather et al., 2022a). Market estimates indicate that the global gelatin market is expected to reach USD 6.7 billion by the end of 2027, with an annual growth rate of 9.29%, driven by the increasing global demand for sustainable functional ingredients (Rather et al., 2022a).

Traditionally, commercial gelatin has been primarily extracted from pig and cattle skins and bones; however, religious and cultural restrictions, alongside health concerns related to the emergence of diseases such as bovine spongiform encephalopathy, have prompted the search for safer and more socially acceptable alternative sources (Abedinia et al., 2017). In this context, studies have focused on exploring alternative collagen sources, including aquatic organisms and poultry (Kaewdang et al., 2016; Mohtar et al., 2011; Mokrejš et al., 2019).

Although fish-derived gelatin is considered a viable alternative, some studies have reported lower gel stability and weaker rheological properties compared to mammalian gelatin, in addition to documented allergic reactions (Al-Nimry et al., 2021). Consequently, poultry industry by-products have emerged as a rich and sustainable source of structural proteins (Santana et al., 2020), with chicken feet in particular being among the most promising by-products for gelatin production (da Almeida et al., 2012).

A study by Esmaeili-Kaliji et al. (2025) demonstrated that gelatin extracted from chicken feet outperforms commercial bovine gelatin in terms of color, gel strength, viscosity, melting point, and amino acid composition, and exhibits a more porous microstructure and higher structural stability. Chicken feet gelatin is characterized by a collagen-rich amino acid profile, including glycine, hydroxyproline, and proline, which play a crucial role in stabilizing the triple-helical collagen structure. Furthermore, this gelatin exhibits higher thermal stability compared to mammalian or fish-derived gelatin (Nik Aisyah et al., 2014; Bichukale et al., 2018).

Chemically, gelatin is a high molecular weight polypeptide resulting from partial thermal hydrolysis of collagen, where heat treatment breaks intramolecular bonds and induces structural and physicochemical changes, forming a gel network capable of water retention (Gál et al., 2020). Melting point and gel strength are among the most important indicators determining the commercial quality of gelatin and its industrial applications (Perez-Puyana et al., 2019).

From a resource utilization perspective, chicken foot skin is a low-cost by-product that remains underexploited in the poultry industry (Miwada et al., 2025a). Liu et al. (2012) indicated that over 40% of claw protein consists of insoluble proteins, primarily collagen, which can generate hydrophobic amino acids capable of donating hydrogen atoms to neutralize free radicals, conferring potential antioxidant properties (Lin et al., 2010; Lee et al., 2012).

Moreover, consuming collagen-rich chicken feet supports skin regeneration and confers multiple health benefits. Chicken foot skin can also be utilized as a functional food ingredient in the form of gels, edible films, or fine packaging applications, enhancing its applicability in advanced food industries (Mulyani et al., 2017; Kim et al., 2017; Miwada et al., 2023).

In the context of bio-packaging, chicken feet gelatin has been developed into composite biodegradable nanofilms when combined with chitosan and zinc oxide nanoparticles, demonstrating efficacy in extending the shelf life of fresh grapes by reducing weight loss, browning index, and controlling bacterial growth (Fatima et al., 2022). Gelatin has also been used with lotus stem starch to produce edible coatings for cherry tomatoes, preserving firmness and reducing weight loss over 15 days (Rather et al., 2022b).

In the field of functional material development, chicken feet gelatin has been combined with chemical compounds to produce a poly(Br-PS)-g-Gelatin polymer, which exhibited antioxidant and antimicrobial activities, thereby expanding its applications in food, medical, and chemical industries (Hashem et al., 2023).

A study by Hussein et al. (2025) indicated that gelatin properties vary with the age of the chicken; the highest yield was achieved at 16 weeks, while maximum gel strength was recorded at 12 weeks. Its addition to yogurt demonstrated effectiveness in preventing whey separation and achieving high sensory acceptance.

Collectively, these findings highlight that chicken feet represent a strategic source of collagen and gelatin, combining economic sustainability, social acceptability, and advanced functional properties. Technically, data indicate the potential to obtain gelatin with thermal and structural properties competitive—and in some aspects superior—to traditional sources. Environmentally, repurposing chicken feet contributes to reducing the ecological burden of slaughterhouse waste within a circular economy framework. From a health and application perspective, the bioactive potential of collagen extracted from chicken feet opens avenues for developing high-value functional and food products.

Nevertheless, further studies are needed to systematically compare chicken feet gelatin with other sources in terms of industrial performance, biosafety, scalability, and economic feasibility. Moreover, optimizing extraction and modification techniques (enzymatic or physical) may be critical for maximizing the functional properties of the final product. Therefore, research investment in chicken feet collagen is not merely an alternative option but represents an integrated strategic approach combining industrial innovation, environmental sustainability, and economic value maximization for poultry by-products.

### Strategic framework for poultry science program in higher education to utilize chicken feet as a promising resource

Maximizing the utilization of poultry industry by-products represents one of the modern strategic directions in sustainable food production systems, amid the global trend toward reducing waste and enhancing the efficient use of animal resources. Despite the significant global expansion in poultry production, chicken feet are still classified in many production systems as a low-value by-product, exploited in limited traditional ways, despite their high content of collagen, gelatin, and bioactive peptides with promising food, pharmaceutical, and industrial applications. This positions them to become a high-value strategic resource within an advanced animal product economy. Despite these potentials, the field of applied research related to the production, processing, preservation, and supply chain development of chicken feet remains emerging and requires further expansion, whether in their direct food applications or in industrial applications based on collagen and gelatin extraction and their functional derivatives. Furthermore, the limited number of integrative studies linking production, processing, storage stability, and efficiency in transportation and marketing represents a scientific and industrial gap that limits the maximization of the economic value of this resource.

Accordingly, rather than presenting a purely conceptual framework, this study proposes a practical implementation model led by universities and research institutions, structured into sequential and interconnected stages. The model begins with the research and knowledge generation stage, where universities conduct applied scientific studies on chicken feet and their bioactive components. This is followed by a technology development stage, which focuses on optimizing extraction and processing techniques for collagen and gelatin. The third stage emphasizes industry integration and practical application, where partnerships with the food and pharmaceutical industries facilitate the transformation of research output into marketable functional products. This is complemented by a capacity-building stage, aimed at developing specialized technical skills and training students and researchers in advanced processing and analytical techniques. Finally, the model incorporates a sustainability and circular economy stage, ensuring the optimal utilization of by-products, minimizing waste, and supporting environmental sustainability while generating measurable economic and societal value. Through this structured approach, the proposed model bridges the gap between academic research and industrial application, ensuring practical applicability, scalability, and long-term impact. The model is implemented through the following interrelated stages:

#### Genetic and production research and development

Genetic and production R&D represents a promising strategic pillar in the transition from traditional exploitation of chicken feet to value-driven production based on functional and bioactive properties. Although the quality of extracted collagen and gelatin has traditionally been associated with downstream processing and manufacturing techniques, the quantity and quality of collagen are influenced by primary biological factors, including breed genetics, growth rates, connective tissue structure, and metabolic efficiency in the bird, aspects that still require systematic in-depth studies to understand and verify these relationships.

From this perspective, poultry science programs at universities and their affiliated research centers can play a crucial role in developing specialized research plans aimed at characterizing commercial and local broiler strains regarding collagen content in feet, its structural and functional properties, and analyzing how it is affected by rearing, nutrition, and environmental conditions. Applications of advanced genomic analysis and AI-assisted selection models represent a promising future research direction rather than established evidence base within the current study. This line of inquiry may be explored in subsequent investigations to identify and validate potential genetic markers associated with collagen characteristics and quality in poultry species. However, such hypotheses remain in their early stages and require extensive experimental validation through well-designed genomic, molecular, and phenotypic studies before being translated into practical breeding or selection programs.

The current study approach enables a gradual shift from the concept of producing chicken solely for meat to an innovative multi-value breeding model, integrating meat production goals with the exploration and maximization of potential industrial value of by-products, supporting the development of a high-quality animal-based gelatin industry founded on sustainable scientific principles.

#### Improvement of extraction and manufacturing processes

Developing techniques for extracting and manufacturing gelatin from chicken feet is an advanced research area that has witnessed notable progress in recent years. Studies have demonstrated its potential for producing edible films and packaging materials with multifunctional properties, including antimicrobial activity, oxidative reduction, and control of moisture and gas transfer. Despite the experimental validation of these applications, their integration into a comprehensive industrial system for poultry by-product utilization remains limited, particularly in aligning extraction technologies with food manufacturing requirements and industrial value chains.

Within this context, the proposed strategic framework aims to transition from isolated research applications to an integrated production model focused on improving extraction efficiency, standardizing functional properties of the extracted gelatin, and developing manufacturing platforms capable of converting it into high-value bio-packaging materials. This approach also requires evaluating the impact of various extraction techniques on the molecular and functional properties of gelatin to ensure its suitability for sustainable industrial production. Consequently, the strategic value of chicken feet extends from a low-value by-product to a sustainable resource for producing biodegradable packaging materials that support sustainability principles, the circular economy, and reduce reliance on conventional plastic polymers.

#### Innovation in food and health products

Innovation in developing food and health products represents one of the most important practical pathways to maximize the added value of chicken skin and feet. Gelatin and bioactive peptides extracted from these by-products allow a shift from traditional uses to designing functional foods that address growing health and nutritional needs across different population groups. Thanks to their high content of essential amino acids and minerals associated with collagen structure, chicken feet gelatin can be used to develop high-protein, easily digestible foods supporting the nutritional needs of children, pregnant women, adolescents, and the elderly, particularly regarding bone and joint health and muscle mass improvement.

It is possible to develop innovative scientific methods to produce biologically active peptides with antioxidant and anti-inflammatory properties, which can be utilized as dietary supplements to support skin and connective tissue health and prevent disorders associated with oxidative stress. At the industrial level, extracted gelatin provides an innovative functional alternative to fat in processed meat products such as sausages and burgers, improving water retention, texture, and sensory perception without increasing fat content. This supports the trend toward producing healthier, low-fat foods while maintaining acceptable sensory quality for consumers.

To achieve this transformation, poultry science programs at higher education institutions play a pivotal role as transitional platforms between academic research and commercial application, developing prototypes for sustainable food and medical products ready for manufacturing and marketing, thereby enhancing universities’ role as engines of bio-food innovation and supporters of a knowledge-based economy.

#### Building academic and technical capacities

Building academic and technical capacities forms a fundamental pillar to ensure sustainable innovation and the development of the value chain for chicken feet and their by-products. This requires integrating advanced programs and specialized training focused on protein and gelatin sciences, as well as food and industrial innovation of by-products, equipping students and researchers with theoretical knowledge and practical skills needed to develop innovative scientific and industrial solutions. In this context, modern approaches to autonomous and technology-enhanced learning play a critical role in strengthening training systems and improving knowledge transfer efficiency, particularly in specialized scientific domains (Wu et al., 2024).

These programs also prepare skilled personnel capable of designing advanced research experiments, analyzing functional properties of gelatin and proteins, and exploring new food and medical applications. Additionally, organizing joint workshops with food and pharmaceutical companies serves as an effective tool for transferring knowledge and modern technologies from academic laboratories to the industrial environment, enhancing university-industry collaboration. These workshops allow students and researchers to observe the latest innovations, understand real-world manufacturing challenges, and gain hands-on experience applying research findings, enhancing their capacity to develop sustainable, market-ready food and medical products, while strengthening universities’ positions as drivers of industrial innovation and development.

#### Partnerships and marketing

Partnerships and marketing form a central element in transforming chicken feet and their by-products into strategic resources with high economic value. Poultry science programs in universities can play an active role in linking scientific innovation with commercial opportunities, including collaboration with national and international companies to develop export-ready products, leveraging industry expertise in manufacturing, and ensuring compliance with quality standards and international certifications, thereby enhancing the global competitiveness of these products.

Establishing an integrated marketing network serves as a strategic tool to support exports of chicken feet and locally produced gelatin, promoting products, facilitating distribution channels, connecting producers with potential buyers, and strengthening communication with industrial and commercial partners. Additionally, analyzing global demand and studying target markets, such as China and Southeast Asia, is essential for guiding research and industrial production to align with consumer requirements and market trends. Ensuring that innovation meets the actual needs of major markets contributes to maximizing economic returns and industrial sustainability of these vital by-products.

### Implications of implementing the strategic framework for poultry science programs in universities

Implementing this strategic framework for poultry science programs in universities represents a pivotal step toward transforming chicken feet and their by-products into high-value scientific, industrial, economic, and health resources. It enables universities to enhance research and innovation, develop specialized academic and technical capacities, link research outcomes with industrial applications, and open new market opportunities for functional food products, health supplements, and sustainable packaging materials. This framework also contributes to environmental sustainability and the circular economy by reducing animal waste, optimizing the use of all by-products, improving food quality and safety, and enhancing the ability of local and international industries to leverage these resources. Thus, the framework establishes a culture of long-term innovation and applied research and reinforces the role of universities as a key driver of scientific, economic, and social development in the poultry sector.

#### Academic and research implications

The academic and research dimension represents one of the most significant gains resulting from the implementation of the strategic framework in poultry science programs at higher education institutions. It opens the door to pioneering scientific innovation by exploring new research areas in nutrition, genetics, and biochemistry related to collagen and gelatin, thereby enhancing understanding of the functional properties of by-products. The framework also contributes to developing research capacities for students and researchers by training specialized personnel in protein extraction and analysis techniques for use in manufacturing sustainable food and medical products, raising the practical and academic readiness of institutions.

The current study focus facilitates the generation of new knowledge that enriches the scientific literature on breed improvement, high-quality gelatin production, and sustainable utilization of by-products, in addition to promoting collaboration through strategic partnerships with research and industrial institutions to transfer knowledge and modern technologies. Consequently, poultry science programs become vital platforms for innovation and research excellence at both local and global levels.

#### Economic and industrial implications

The economic dimension of implementing this strategic framework represents a significant opportunity for industrial entities to maximize the financial value of chicken feet, which are currently underutilized by-products. By converting these feet into marketable products such as high-quality gelatin, health-promoting peptides, and functional foods, new income streams can be created, enhancing industrial profitability. This approach also contributes to waste reduction and decreases environmental costs by fully utilizing this vital part of the bird instead of discarding it as waste.

Moreover, innovation in using chicken feet opens new market opportunities and encourages the food and pharmaceutical industries to adopt sustainable production practices, enhancing the integration of scientific utilization with industrial application of the economic value of these secondary resources.

#### Health and societal implications

Implementing this strategic framework contributes to public health by transforming chicken feet into functional food products rich in collagen and bioactive peptides that support skin, joint, bone, and cardiovascular health. These products also provide comprehensive dietary alternatives that can be targeted toward specific population groups, such as pregnant women, adolescents, and the elderly, to enhance functional nutrition and improve quality of life.

From a societal and environmental perspective, the optimal use of by-products reduces animal waste and residuals from conventional production, contributing to the conservation of natural resources, reinforcing environmental sustainability principles, and creating an integrated model linking academic research in poultry science programs, industrial application, and community well-being.

#### Long-term strategic implications

The long-term strategic impact of implementing this framework in poultry science programs represents an opportunity to enhance the role of universities as primary drivers of innovation, where scientific research outcomes are transformed into products applicable in industrial and societal contexts. This reinforces the integration between academic knowledge and the needs of industry and society.

The framework also accelerates the development of a sustainable bio-economy by strengthening the linkage between education, research, and industry according to sustainability and circular economy standards, ensuring optimal utilization of secondary resources such as chicken feet. Furthermore, it establishes a culture of industrial innovation within universities, inspiring students and researchers to design and develop innovative functional products, thereby cultivating a new generation of professionals capable of transforming available biological resources into sustainable food and industrial solutions with high economic value.

### Challenges to the sustainability and utilization of chicken feet in local and global markets

The utilization of chicken feet and secondary products represents a significant economic opportunity; however, the success of this strategic model directly depends on the ability to address market and trade challenges at multiple levels. Implementing this initiative is not limited to production and industrial technologies alone but also involves ensuring continuity in supply chains, achieving consumer and food industry acceptance, addressing economic fluctuations and regulatory frameworks, and adapting to the requirements of local and international markets. Consequently, studying the determinants of trade and market challenges is a necessary step to understand the operational, economic, and social barriers that may hinder the transition from production capacities to sustainable economic benefits and the alignment of a comprehensive value chain strategy.

#### Challenges in supply chain sustainability and industrial integration

Supply chain sustainability and industrial integration represent key operational challenges for the practical implementation of the strategic framework. Although industrial capacities exist, the success of utilizing chicken feet depends on ensuring a regular and stable flow of secondary products through an integrated system connecting farms, slaughterhouses, processing and manufacturing units, and research centers. Poor coordination or the absence of unified logistical management systems leads to variability in raw material quality and differences in biological characteristics, which negatively impact the consistency of extracted products and limit the ability to plan production and long-term industrial expansion.

Furthermore, the misalignment of production and processing schedules across different segments of the value chain may increase operational waste and costs, making institutional integration and efficient supply chain management essential conditions for ensuring the economic and operational sustainability of this industrial model.

#### Challenges in market acceptance and scaling applications

Market acceptance and scaling the application of products derived from chicken feet represent a central challenge to achieving full economic utilization, whether marketing them for direct consumption as food products or using their bioactive components, such as collagen and gelatin, as functional ingredients in other food products. Despite the readiness of production technologies, the adoption of these products remains closely linked to the degree of acceptance by consumers and the food industry, particularly in light of traditional perceptions associated with animal by-products.

Overcoming this challenge requires reintroducing chicken feet and their derivatives as high-value nutritional and functional sources supported by scientific evidence, alongside developing marketing and awareness strategies highlighting their health and nutritional benefits and their role in supporting sustainability and reducing food waste. Expanding industrial use also necessitates demonstrating technical efficiency and functional stability when incorporated into processed foods, thereby enhancing the confidence of food companies and supporting the transition from limited-use products to established components within modern food manufacturing systems.

#### Global competition for chicken feet and its impact on sustainability

Chicken feet are experiencing increasing global competition due to their diverse applications across different markets. They are widely consumed as a human food source in many Asian countries such as China, while also serving as a key ingredient in feed for fur-bearing animals in some European countries (Urlings et al., 1993; Lyhs et al., 2019). In addition, their growing use as a source for extracting bioactive compounds such as collagen and gelatin is further driving global demand and intensifying pressure on supply chains. This multi-purpose competition may affect the availability of this raw material, the stability of its prices, and its equitable distribution across different sectors, thereby posing challenges related to efficient resource management and sustainability. Accordingly, achieving the optimal utilization of chicken feet requires the adoption of balanced policies and integrated strategies that consider global competition and promote the sustainable and equitable use of this valuable resource, serving both food security and industrial applications.

#### Economic and trade challenges

Fluctuations in global markets represent one of the most prominent economic challenges that may affect the sustainability of investing in chicken feet and their secondary products. International demand for chicken feet and animal-derived gelatin is influenced by multiple factors, including trade policies, health restrictions, regulatory changes, and poultry sector epidemics, such as avian influenza outbreaks, which may lead to sudden disruptions in export flows and supply chains.

Additionally, growing competition with alternative sources of gelatin, whether plant-based or marine-derived, places pricing pressures on animal-derived products and necessitates the development of competitive advantages based on quality and advanced biofunctional properties. Institutions also face challenges associated with volatility in raw material prices, energy, and transportation costs, which directly affect the economic feasibility of extraction and manufacturing operations, especially during industrial scaling phases. This challenge is further compounded by the need to build business models capable of balancing high added value with acceptable production costs.

Despite the promising industrial potential, the chicken feet market remains relatively specialized. According to Tridge (2020), new suppliers face several challenges, including limited local demand in many exporting countries, which reduces the number of specialized farms capable of processing these products. Targeting major markets, such as China, requires aggregating large quantities over periods that may extend for several months, which poses a challenge given the short shelf life (2–3 months). Additionally, varying health and regulatory certification requirements for each importing market represent a regulatory and logistical barrier.

## Conclusion

This study highlights chicken feet as a highly underutilized poultry by-product with significant nutritional, functional, and industrial value. Through an integrated framework based on systematic review, data synthesis, and strategic interpretation, the study demonstrates that chicken feet represent a valuable biological resource rather than mere processing waste. The findings confirm their richness in collagen, gelatin, bioactive peptides, and essential minerals, along with functional properties that support human health, positioning them as a promising raw material for functional foods, dietary supplements, and bio-based packaging applications. Moreover, the study underscores the evolving perception of chicken feet, which are increasingly recognized as valuable inputs in modern food processing rather than simply low-value by-products. The study also emphasizes the strategic role of poultry science programs in higher education as key drivers of innovation and industry integration, contributing to transforming this resource into economically and environmentally sustainable products. In conclusion, this study provides a concise strategic perspective that supports repositioning chicken feet within a circular and sustainable poultry economy, with the potential to enhance resource utilization, support innovation, and contribute to food system sustainability.

## Funding

This work was supported by the Deanship of Scientific Research, Vice Presidency for Graduate Studies and Scientific Research, King Faisal University, Saudi Arabia (Grant No. KFU261105).

## CRediT authorship contribution statement

**Farid S. Nassar:** Writing – review & editing, Writing – original draft, Visualization, Validation, Supervision, Software, Resources, Project administration, Methodology, Investigation, Funding acquisition, Formal analysis, Data curation, Conceptualization.

## Disclosures

The author declares that there are no conflicts of interest regarding the publication of this paper.
